# Evidence for Metabolic Provisioning by a Common Invertebrate Endosymbiont, *Wolbachia pipientis*, during Periods of Nutritional Stress

**DOI:** 10.1371/journal.ppat.1000368

**Published:** 2009-04-03

**Authors:** Jeremy C. Brownlie, Bodil N. Cass, Markus Riegler, Joris J. Witsenburg, Iñaki Iturbe-Ormaetxe, Elizabeth A. McGraw, Scott L. O'Neill

**Affiliations:** School of Biological Sciences, The University of Queensland, St. Lucia, Queensland, Australia; The Pennsylvania State University, United States of America

## Abstract

*Wolbachia* are ubiquitous inherited endosymbionts of invertebrates that invade host populations by modifying host reproductive systems. However, some strains lack the ability to impose reproductive modification and yet are still capable of successfully invading host populations. To explain this paradox, theory predicts that such strains should provide a fitness benefit, but to date none has been detected. Recently completed genome sequences of different *Wolbachia* strains show that these bacteria may have the genetic machinery to influence iron utilization of hosts. Here we show that *Wolbachia* infection can confer a positive fecundity benefit for *Drosophila melanogaster* reared on iron-restricted or -overloaded diets. Furthermore, iron levels measured from field-collected flies indicated that nutritional conditions in the field were overall comparable to those of flies reared in the laboratory on restricted diets. These data suggest that *Wolbachia* may play a previously unrecognized role as nutritional mutualists in insects.

## Author Summary

*Wolbachia* are bacteria that infect millions of insect species worldwide. *Wolbachia* aren't infectious, but are maternally inherited symbionts passed from mother to offspring. To infect a host population, *Wolbachia* behave as reproductive parasites and alter the host reproductive system in a manner that increases infected female reproductive success. Some strains of *Wolbachia*, however, cannot manipulate their host's reproductive systems—yet they can successfully infect insect populations. How is this possible? Here we show that a *Wolbachia* strain that naturally infects *Drosophila melanogaster*, and induces very low levels of reproductive parasitism, can also act as a nutritional mutualist. When *D. melanogaster* flies were reared on normal diets, we observed no cost or benefit associated with the *Wolbachia* infection. But, if we reared flies on diets containing either very low or high amounts of iron, *Wolbachia*-infected flies produced more eggs than uninfected flies. As wild-caught flies contain low amounts of iron, our results suggest that flies in the wild should benefit from their *Wolbachia* symbiont.

## Introduction

*Wolbachia pipientis* is arguably the most abundant endosymbiont in the insect world [1]–[4]. It is strictly maternally inherited and has evolved a number of mechanisms, broadly classed as reproductive parasitism, to facilitate its invasion into host populations. The most common of these traits is termed cytoplasmic incompatibility (CI), which is a form of early embryonic developmental arrest seen in the offspring of uninfected female insects that have been mated to an infected male. CI can also be seen in cases where *Wolbachia* infected females have mated to males carrying an unrelated *Wolbachia* strain [5],[6]. CI results in *Wolbachia* infected females possessing a reproductive advantage over uninfected females and as a result *Wolbachia* is able to invade host populations [7],[8].

CI is considered to be the major driving force behind *Wolbachia* invasion. This paradigm, however, is based primarily on experimental work with a limited number of *Wolbachia* strains that induce strong CI in *Drosophila simulans* [7]–[9]. A number of *Wolbachia* strains, including all of those recovered from *D. melanogaster*, induce very weak and variable CI especially under field conditions [10]–[12]. Theoretical work indicates that such weak CI is unlikely to be sufficient to drive a *Wolbachia* invasion in the field. Moreover, strains of *Wolbachia* have been indentified in *D. simulans* that induce no CI at all, yet these strains have managed to invade host populations [12],[13]. Alternatively, these strains may represent defective CI inducing strains that have lost the ability to induce CI and represent a snapshot of a *Wolbachia* infection that in time will be lost [14]. In the absence of strong CI induction the most obvious explanation for the ability of these strains to invade would be that they do not act as reproductive parasites at all, but possibly serve some mutualistic function for the insect. *Wolbachia* infections in *Drosophila melanogaster* have been observed to act positively upon non-reproductive fitness traits, such as the extension of adult lifespan or protection against viral and fungal pathogens [15]–[17]. Yet despite considerable efforts to find a positive fecundity benefit for *Wolbachia* infected *Drosophila melanogaster*, none has been identified [18].

*Wolbachia* also infect filarial nematodes, where the bacterium is an obligate mutualist required for successful reproduction and development of the worm [19],[20]. Analysis of the full genome sequence of the worm, *Brugia malayi* revealed that it lacked a complete biosynthetic pathway for both heme and riboflavin [21]. In contrast, the much reduced genome of its infecting *Wolbachia* strain, *w*Bm contained a complete suite of genes for both pathways [22]. Genes that encode components of the heme biosynthetic pathway were subsequently shown to be under diversifying selection in *w*Bm, providing further support for the hypothesis that this pathway may be a key point of interaction in the association and offering a potential explanation for the basis for the obligate mutualism [22]. Positive selection was also identified on genes in the same pathways in the genome of the *Wolbachia* strain, *w*Mel that infects *D. melanogaster* [23], raising the possibility that the bacterium may play a role in metabolic provisioning in insects as well as nematodes [24].

Although insect hosts are not dependent upon *Wolbachia* for heme biosynthesis, the microbe could supplement host stores or play a role in iron homeostasis. Iron is an essential micronutrient required for a diverse range of metabolic processes [25]–[27] including maturation and development of the insect egg [28]. Iron also varies in the environment [26] and hence is likely to be variable in the diet of wild insects. Here we examine how the presence of *Wolbachia* infection alters fitness of the model insect host, *Drosophila melanogster* when reared under varying levels of dietary iron to test the hypothesis that *Wolbachia* may function as a nutritional mutualist as well as a reproductive parasite in insects.

## Results

**Figure 1:**
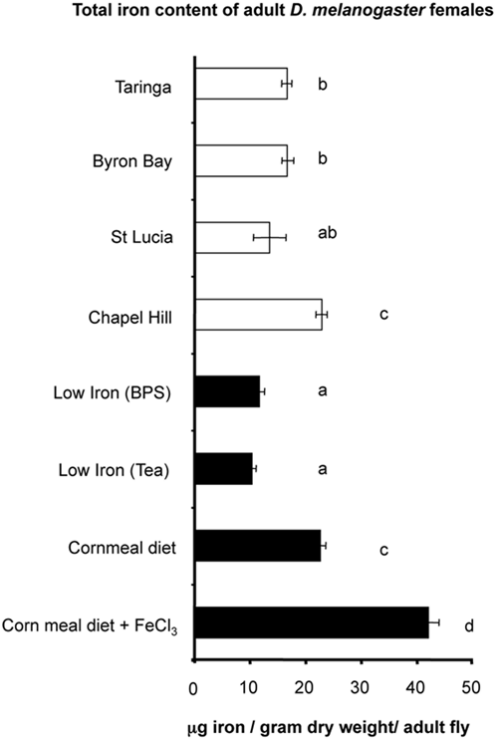
Total iron content of adult *D. melanogaster* females as determined by inductive coupled plasma mass-spectrometry. Field collected flies are represented by white bars and lab reared flies by black bars. Flies reared on cornmeal fly diet contained approximately half the amount of iron as flies reared on high iron food, and approximately twice the amount of flies reared on low iron diets. All observations of lab-reared flies are statistically different from each other (t test; p<0.0001; groups a, c and d). The total iron content for three of the four field caught fly populations were significantly lower than those reared on normal food, where no *Wolbachia* fecundity advantage was observed (t test; p<0.0001; group b). The total iron content of flies collected from the St Lucia field site (t test; p>0.9999) was not significantly different from flies reared under low iron conditions where *Wolbachia*-associated fecundity benefits were observed. Standard error bars are indicated. Analysis was performed on pools of 10 adult female flies. A total of 10 pools were examined for each of the four defined diets; 4 pools of flies were examined for field-collected flies.

The total amount of iron within *D. melanogaster* was responsive to our dietary manipulations as measured by mass-spectrophotometry. Flies reared on high iron diets contained approximately twice as much total iron as those reared on cornmeal diet (Figure 1). Flies reared on either tea or BPS diets had approximately half the amount of total iron compared to those reared on cornmeal diet. The presence of *Wolbachia* did not influence the total iron content of adult *D. melanogaster* as both infected and uninfected fly lines were estimated to have similar total iron contents (data not shown). The total content of eight other biologically relevant metals (see Materials and Methods) did not change in response to the altered diets; the only metal that was responsive to the modified diets was iron.

The total iron content of field adult female flies collected from four locations in and around Brisbane, Australia, was also determined. The total iron content of adult female flies from three of the four collection sites were similar to that determined for flies reared on low iron diets (Taringa, Brisbane; St Lucia, Brisbane; and Byron Bay; Figure 1). At one location (Chapel Hill, Brisbane), the total iron content was higher than other locations, and was similar to that observed for flies reared on standard cornmeal fly diets. Not surprisingly total iron content of wild flies was different at each location, but in general iron content of flies taken from the field was lower (t test; p<0.0001) than that of lab reared flies on cornmeal diet and more similar to lab flies reared on restricted diet.

**Figure 2:**
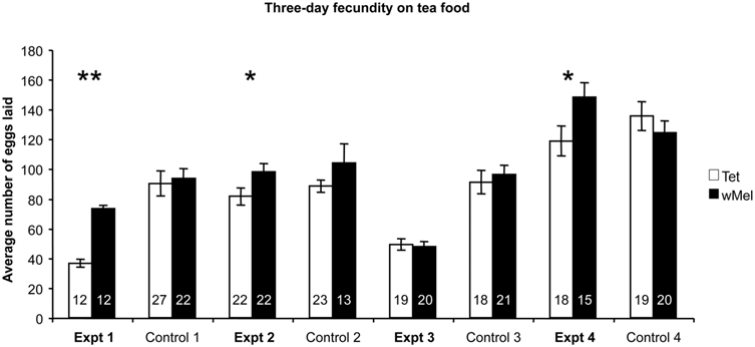
Mean fecundity measures of female *D. melanogaster* reared on low iron food (tea). The total number of eggs laid by a single female was counted over a three-day period and the average calculated. Standard error bars are indicated; replicate numbers are noted within the columns. Uninfected females are denoted by an open bar, *Wolbachia*-infected females by a filled bar. Female flies reared on cornmeal fly diet are described as “Control.” Mean fecundities that are significantly different are denoted by * (p<0.05; ANOVA) or ** (p<0.001; Mann-Whitney U Test).

**Figure 3:**
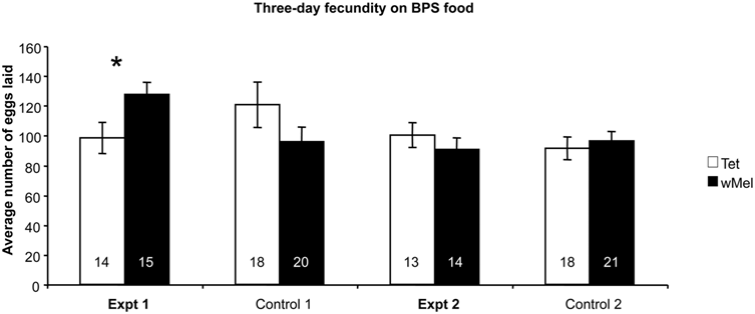
Mean fecundity measures of female *D. melanogaster* reared on low iron food (BPS). The total number of eggs laid by a single female was counted over a three-day period and the average calculated. Standard error bars are indicated; replicate numbers are noted within the columns. Uninfected females are denoted by an open bar, *Wolbachia*-infected females by a filled bar. Female flies reared on cornmeal fly diet are described as “Control.” Mean fecundities that are significantly different are denoted by * (p<0.05; ANOVA).

To investigate if *Wolbachia* could provide a fecundity benefit to *D. melanogaster* in a low iron environment, two fly lines both derived from the same genetic background were used [11]. The first line was infected with *w*Mel, the second was uninfected due to the prior application of antibiotics. *Wolbachia* had no effect on the fecundity of *D. melanogaster* when reared on cornmeal fly diets. (Controls 1–4; Figure 2). In contrast, when *D. melanogaster* females were reared on low iron diets due to the addition of black tea, *Wolbachia* conferred a fecundity advantage in three of four independent experiments. In one experiment (Figure 2, Expt 3) no significant difference in fecundity was observed. Where a fecundity advantage was observed, *Wolbachia* infected *D. melanogaster* females laid on average 20% more eggs than uninfected females (Expt 2 and 4; Figure 2), and for one experiment a 50% advantage was observed (Expt 1; Figure 2). In no experiments was there a fecundity reduction in the presence of *Wolbachia*. In addition to using black Tea as an iron chelating agent bathophenanthroline disulfonate (BPS) was used to specifically chelate iron(II) [29] in two independent experiments. Again variable results were obtained with a 20% fecundity advantage seen in one experiment and no difference seen in a subsequent experiment (Figure 3). To ensure the Tea or BPS diets were efficiently chelating iron we measured the total iron content of flies that showed no fecundity advantage and compared these to flies that did. We observed no statistical difference among these treated flies (t test; p>0.9999) and conclude the diets used successfully reduced the total iron of *D. melanogaster* flies.

**Figure 4:**
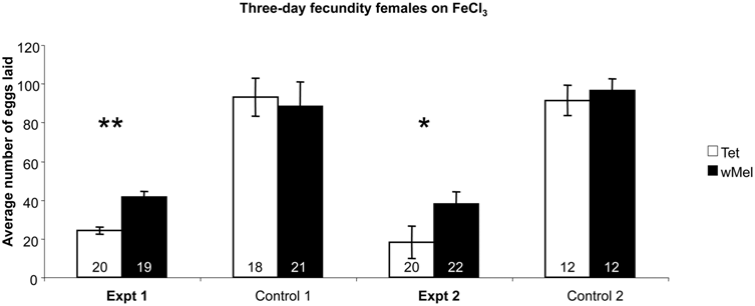
Mean fecundity measures of female *D. melanogaster* reared on high iron food (FeCl_3_). The total number of eggs laid by a single female was counted over a three-day period and the average calculated. Standard error bars are indicated; replicate numbers are noted within the columns. Uninfected females are denoted by an open bar, *Wolbachia*-infected females by a filled bar. Female flies reared on cornmeal fly diet are described as “Control.” Mean fecundities that are significantly different are denoted by *(p<0.05) or ** (p<0.001; Mann-Whitney U Test).

When *D. melanogaster* were reared on diets that contained high levels of iron, due to the addition of FeCl_3_ we observed a significant reduction in fecundity for both infected and uninfected fly lines relative to flies reared on cornmeal diets. However, the presence of *Wolbachia* in flies on high iron diets resulted in significant gains in fecundity in two independent experiments (p<0.05 and p<0.001 Mann-Whitney U; Figure 4). As the FeCl_3_ diet contains both higher concentrations of iron as well as chloride, we reared flies on NaCl diets to determine if the *Wolbachia* associated effects were due to the addition of iron and not chloride. We observed no fecundity difference between infected and uninfected *D. melanogaster* females when reared on a diet rich in chloride (data not shown). Therefore we conclude that the fitness benefits conferred by *Wolbachia* were in response to the high iron content.

Assessments of *Wolbachia* effects on male fertility in response to changes in dietary iron were performed using *Wolbachia* infected and uninfected BNE lines reared on Tea, BPS and FeCl_3_ diets. In no experiment did we observe a cost or benefit to male fertility associated with *Wolbachia* infections (data not shown), and conclude that *Wolbachia* only benefits female fecundity and not male fertility during periods of iron deficiency or overload.

## Discussion

Metabolic provisioning of hosts by endosymbionts is commonly observed in obligate associations [30]. *Wolbachia* strains that infect filarial nematodes are one such example, and are thought to provide their host with essential vitamins, nucleotides and cofactors, including heme [22]. These same biosynthetic pathways exist in insect *Wolbachia*, and evolutionary analyses have previously identified signatures of positive selection on pathway genes [23]. Given the multiple predictions of heme and iron as potential interaction points for *Wolbachia* and their hosts, we experimentally determined if *Wolbachia* could influence iron homeostasis and female fecundity in an insect host.

*Wolbachia* had no effect on *Drosophila melanogaster* fecundity when reared on cornmeal diets, consistent with previous observations [31]. In contrast, *Wolbachia* did provide a significant fecundity benefit to female *Drosophila* when subjected to low or high iron environments in the majority of experiments conducted. This is the first report of a *Wolbachia* conferred compensatory effect during periods of nutritional stress or deficiency to an insect host. The observed variability in fecundity measures is consistent with previous experiments in *D. melanogaster*, which have shown that laboratory measurements of fecundity are highly sensitive to local assay conditions, and are notoriously difficult to replicate even under controlled laboratory conditions [32]–[34].

Given the observed *Wolbachia* fecundity advantage in perturbed iron environments and the observed low iron content of flies from the wild, it is likely that the results of the laboratory experiments reported here may have ecological relevance, providing a variable but positive fitness benefit to *Wolbachia* infected flies across a range of environments. Previous studies have shown that if *Wolbachia* can simultaneously induce cytoplasmic incompatibility and increase female fecundity, the rate at which *Wolbachia* invades naïve host populations is increased [35].

Benefits observed under high iron conditions, while not ecologically relevant based on the estimates of total iron in field caught flies, are interesting mechanistically. Increases in dietary iron result in an increase in oxidative stress for most insects [36], and in our experiments severely reduced the fecundity of both infected and uninfected females. The fecundity cost incurred by infected females was, however, reduced relative to uninfected females suggesting that *Wolbachia* might provide protection against oxidative stress.

## Materials and Methods

### Fly rearing

The *Drosophila melanogaster* strain BNE was derived from field caught female flies from Brisbane, Australia, and is described in detail elsewhere [11]. In brief, field caught flies were treated with tetracycline to remove endosymbiont bacteria and a *w*Mel infection introgressed by crossing to yw^67c23^ females. Subsequent offspring were backcrossed with males derived from the original field collection for a minimum of five generations to re-establish the original BNE genetic background. All flies were maintained at ∼25°C on a 12/12hr light/dark schedule throughout the study. Tetracycline treatments were performed as described previously [37] to generate a genetically identical fly line that lacked the *Wolbachia* infection. To reconstitute gut flora, stock bottles containing cornmeal fly diet were seeded with non-tetracycline treated males for a period of three days. These males were then excluded from the diet and newly emerged tetracycline treated adult flies allowed to mate and lay eggs on the diet. Assessments of fecundity were performed at least three generations post tetracycline treatment and reconstitution of gut flora to minimise maternal or grandmaternal mitochondrial effects [38],[39]. To minimize genetic drift between these fly lines, approximately every 10 generations reciprocal crosses (BNE-wMel Female × BNE Tet male; BNE Tet female × BNE-wMel Male) were performed using one-week-old adults. Fly lines were reared on four types of diets. Cornmeal fly diet was made from yellow corn meal medium (Sigma). Two low iron diets were made by either substituting the water that was used to make up the medium with an aqueous extract of black tea (*Camellia sinensis*; 3 Tetley tea bags infused in 1litre of water for 5 minutes [40]) or through the addition of 20 μM bathophenanthroline disulfonate (BPS; Sigma) to melted cornmeal fly diet at 65°C. In both instances the amount of available iron to the developing *Drosophila* larvae was reduced by iron chelating agents [41],[42]. A high iron diet was made by the addition of a FeCl_3_ solution to the cornmeal fly diet to a final concentration of 10mM [42]. In a single experiment (Female fecundity: n = 30 Wolbachia-BNE and n = 28 Tet-BNE individuals; Male fertility: n = 20 Wolbachia-BNE and n = 21 Tet-BNE individuals) cornmeal fly diets supplemented with 30mM NaCl were generated as a control to test if the addition of chloride ions from FeCl_3_ could influence fecundity.

### Reproductive analyses

Females were allowed to lay eggs onto molasses/agar plates in the absence of yeast. First instar *D. melanogaster* larvae were introduced to vials containing modified or cornmeal fly diets at low densities (50–80 larvae) and reared to adulthood at 25°C. Virgin males and females were collected and maintained separately on the same diet for a period of three days. Individual crosses among males and females of identical infection status were allowed to mate once within a 60-minute window. Once mating was complete, males were discarded and mated females allowed to oviposit onto molasses plates seeded with yeast for a period of three days. A new plate was introduced every 24 hours and the total number of eggs laid was scored. To determine the impact of *Wolbachia* infection on female fecundity under iron limitation or overload, females reared on modified diets were mated with males reared on cornmeal diet. The reciprocal cross permitted assessment of male fertility. Once the total number of eggs laid over the three-day period had been scored, comparisons of fecundity between *Wolbachia* infected or uninfected *Drosophila* were made using parametric (ANOVA) or non-parametric (Mann-U Whitney) tests where appropriate.

### Iron concentration analysis

The total content of biologically relevant metals (manganese, iron, cobalt, nickel, copper, zinc, Cadmium, lead and arsenic) present in flies reared on each of the food types or collected from the field, were determined using inductive coupled plasma mass-spectrometry (ICP-MS) at the Advanced Centre for Isotope Research Excellence at the University of Queensland. The only metal responsive to diet was iron. Pools of 10 female flies were used for each analysis and replicated ten times for lab reared flies or four times for field caught flies. Flies were caught using modified banana traps, such that flies were attracted to the bait but excluded from feeding upon it to ensure that total iron levels were not affected.
